# Comparison of two histopathologic methods for evaluating subcutaneous reaction to mineral trioxide aggregate

**DOI:** 10.4317/medoral.17309

**Published:** 2011-12-06

**Authors:** Sepideh Vosoughhosseini, Mehrdad Lotfi, Monir Moradzadeh, Amirala Aghbali, Saeed Rahimi, Mohammadali Saghiri, Vahid Zand, Masoumeh Mehdipour, Bahram Ranjkesh, Sirvan Doosti

**Affiliations:** 1 DMD, MSc. Associate Professor. Department of Oral and Maxillofacial Pathology, Dental Faculty, Tabriz University (Medical Sciences), Tabriz, Iran; 2 DMD, MSc. Associate Professor, Research Center for Pharmaceutical Nanotechnology, Tabriz University (Medical Sciences), Tabriz, Iran; 3 DMD, MSc. Assistant Professor. Department of Oral and Maxillofacial pathology, Faculty of Dentistry, Tabriz University (Medical Sciences), Tabriz, Iran; 4 DMD, MSc. Associate Professor. Department of Endodontics, Faculty of Dentistry, Tabriz University (Medical Sciences), Tabriz, Iran; 5 BSc, MSc, PhD, Assistant Professor. Department of Dental Materials, Faculty of Dentistry, Azad University of Medical Sciences, Tehran, Iran; 6 DMD, MSc. Assistant Professor, Department of Endodontics, Dental Faculty, Tabriz University (Medical Sciences), Tabriz, Iran; 7 DMD, MSc. Assistant Professor, Department of Oral Medicine, Dental Faculty, Tabriz University (Medical Sciences), Tabriz, Iran; 8 DMD. Private practice, Tabriz, Iran; 9 DMD, Post graduate student, Department of Endodontics, Dental Faculty, Tabriz University (Medical Sciences), Tabriz, Iran

## Abstract

Objectives: One of the most important factors for suitable materials for pulp therapy is biocompatibility. Two histopathologic methods of Cox and Federation Dentaire International (FDI) were used to evaluate inflammation. In Cox method, density of inflammatory cells, tissue reactions like fibrosis, vascular responses like congestion and fibrin extravasation have been used to evaluate inflammatory reactions. The aim of this study was to compare the accuracy of pathologists’ interpretations using two different methods. Study design: Three pathologists observed the degree of inflammation in 225 histopathologic sections. These sections showed inflammation in subcutaneous connective tissue of rats adjacent to polyethylene tubes, filled with white or gray mineral trioxide aggregate. Empty tubes served as controls. Samples were harvested after 7-, 15-, 30-, 60-, and 90-days. All pathologists examined the sections under a light microscope (Carl Zeiss, Oberkochen, Germany) at ×400 magnifications. Chi-Square test was used to evaluate the difference between inflammation grades when one pathologist used two methods. Cohen’s Kappa value was used to measure agreement of three pathologists to recognize the degrees of inflammations when using one of the methods. Results: There were no significant differences between the two methods when one of the pathologist used these methods to report the degree of inflammation (p=0.054). However, two other pathologists reported significant differences between two methods (p=0.005, p=0.001). In the FDI method, there was an acceptable agreement between first and second, and first and third pathologist in terms of the degree of inflammation, and intermediate agreement existed between the second and third pathologist. With the Cox method, no agreement among the pathologists could be found. Conclusion: The results of three pathologists in terms of rating inflammation with the FDI method showed better agreement than with the Cox method. Therefore, FDI method is more reliable than the Cox method to evaluate inflammation.

** Key words:** Biocompatibility, connective tissue, infammation, mineral trioxide aggregate.

## Introduction

Two methods of microscopic evaluation have been used in rat connective tissue to evaluate biocompatibility of dental materials. The first one was described by Cox et al (1) in 1996. In this method, density of inflammatory cells, tissue reactions like fibrosis, vascular responses like congestion and fibrin extravasation have been used to evaluate inflammatory reactions. Another method is based on Federation Dentaire International (FDI) recommendation in which inflammatory cells count in different areas of microscopic sections (2,3).

Mineral Trioxide Aggregate (MTA) is one of the most common multipurpose materials that are used in endodontics (4). Many studies have evaluated the biocompatibility of MTA in subcutaneous connective tissue of rats. A few studies (5-9) have evaluated the biocompatibility of materials in subcutaneous connective tissue of rat using Cox method. However, some researchers have used FDI method (10-12). Shahi et al (5) used Cox method and has reported that inflammatory reaction to white MTA(WMTA) was significantly more than gray MTA (GMTA) after one week, but they were similar at next intervals. However, Vossoughhosseini et al (11), using FDI method demonstrated the similar inflammatory reactions to W and GMTA in all intervals. The different results might be related to two different histopathologic methods. Therefore, it seems that using a reliable histopathologic method is necessary to evaluate tissue sections. In fact, there are not any studies that compare these two methods. Thus, the aim of this study was to compare the consistency of the results which were obtained from pathologists when using two different methods of FDI and Cox to evaluate biocompatibility of W and GMTA.

## Material and Methods

Fifty male, 2- to 3-month-old Wistar albino rats weighting 250 ± 30 g were used in this study. All ethical and human criteria contained in Tabriz University of Medical Sciences were observed in the different stages of the project. The following materials were examined: Group 1, tooth colored mineral trioxide aggregate (white MTA) (Tooth colored formula, Dentsply, Tulsa Dental, Tulsa, OK, USA); group 2, ProRoot MTA (gray MTA) (Dentsply, Tulsa Dental, Tulsa, OK, USA). Animals were anesthetized with diethyl-ether (Pars chemie, Tehran, Iran) using the chamber-induction method. Three separate 2-cm incisions were made on the back of the rats at least 2 cm away from each other. Freshly mixed materials were prepared according to the recommendation of the manufacturer and were placed in sterile polyethylene tubes with a 1.1-mm inner diameter and 8-mm length and were immediately implanted subcutaneously in two separate incisions. An empty polyethylene tube was implanted in the third incision in each animal as a control in 7-, 15-, 30-, 60-, and 90-day intervals. Rats were euthanized by administrating a high dose of diethyl-ether in an induction chamber. The tubes and surrounding tissues were removed in block and fixed in 10% buffered formalin solution for 2 weeks. Sections of 5µm of tissue were made longitudinally through the midline of the tubes and stained with hematoxylin and eosin. 

Three pathologists with ten years of experience without any knowledge about materials and the results of each other evaluate the inflammatory reactions in microscopic fields adjacent to tested materials at the end of the tubes under a light microscope (Carl Zeiss, Oberkachen, Germany) at 400×magnifications. An average value for each specimen was obtained from 4 separate areas. At first all microscopic slides (600) were coded from 1 to 600 and evaluated by FDI methods. In this method, the inflammatory reactions were categorized as 0, none (without inflammatory cells); 1, mild (inflammatory cells ≤25); 2, moderate (25-125 inflammatory cells); 3, severe (more than 125 inflammatory cells) (7). At the second step, the same three pathologists evaluated the inflammation according to Cox and Robin’s criteria (5) that is based on accumulation of acute and chronic inflammatory cells, fibrin deposits, tissue edema, and vascular congestion. The inflammatory reactions were classified as follows: Grade I, scattered chronic inflammatory cells; grade II, infiltration of inflammatory cells, and wavy collagen fiber deposits and fibrosis; grade III, dense infiltration of inflammatory cells, limited areas of tissue edema and vascular congestion; grade IV, very dense infiltration of acute and chronic inflammatory cells, widespread edematous areas and vascular congestion along with fibrin deposits. The chi-square test was used for statistical analysis of the consistency of the results which were obtained from 2 methods by each pathologist and the “kappa coefficient of agreement” test was used to define the pathologists’ agreement in two different methods.

## Results

There was not any significant difference between FDI and Cox methods when the grades of inflammation were scored by the first pathologist (p= 0.054). However, the grades of inflammation were significantly different from each other when second or third pathologist used these two methods (p= 0.005 and p= 0.001 respectively).

To determine interrater reliability between the three pathologists, we calculated the Cohen kappa (k). Cohen kappa is a measure of the extent to which two (or more) raters agree on a number of categoric outcomes corrected for chance agreement. Kappa values range from 0.0 (no agreement) to 1.0 (perfect agreement), with k ≥ 0.75 denoting excellent reliability (13).

Results indicated that in FDI methods, agreement among pathologists 1 and 2, 1 and 3 and 2 and 3 was acceptable (k = 0.74, k = 0.69 and k = 0.52 respectively), and statistically was significantly different from no agreement (p = 0.0005). However, in Cox method, there was weak agreement among pathologist 1 and 2, 1 and 3 and 2 and 3 (k = 0.23, k = 0.12 and k = 0.13 respectively) (p= 0.0005, p= not significant and p= 0.02 respectively).

## Discussion

Biocompatibility is one of the most important factors to select root-end filling material (14). There are four common methods for evaluating of biocompatibility of root-end filling materials (5).

1) Cytotoxicity evaluation.

2) Subcutaneous implantation.

3) Intraosseous implantation.

4) Clinical and radiographic evaluation of human periapical tissue reaction.

Implantation of tubes which contain tested materials in rat’s connective tissue is a common method to evaluate biocompatibility. This method was introduced by Torneck et al (15) in 1962 and was approved by Olsson et al (16) in 1981. In this method, materials are placed in sterile polyethylene tubes and are implanted subcutaneously in rat’s connective tissue. Materials and surrounding tissues are harvested in appropriate intervals and stained with hematoxylineosin and observed under a light microscope.

Histopathologic evaluations of inflammatory reactions are one of the most sensitive stages of evaluating biocompatibility of materials in subcutaneous implantation. 7-, 15-, 30-, 60- and 90- day intervals were used in this study to evaluate short- and long - term inflammatory reactions. In other words, different histophatologic features can be observed under the light microscope when short- and long- term evaluation are used. Therefore, the agreement among pathologists can be evaluated in long- and short- term periods. For instance, congestion of the blood vessels is expected to see in short- term evaluation and disappear later which is a good sign of subsiding inflammatory reactions (15). Moreover, collagen fibers are thicker in microscopic slides that belong to long- term specimens than short- term ones (15). Thus, it seems that comparing the inflammatory reactions in different intervals is not a hard job. As many studies that have focused on inflammatory reactions following subcutaneous implantation of W and GMTA reported descending trend when coming from short- to long- term intervals (5,11,12,14).

Indeed, interpretation of inflammatory reactions among implanted materials is a big challenge. In Cox’s method, congestion of blood vessels is a feature of inflammation (1). According to this study, the detection of blood vessels in microscopic views occurred in less than 10% of slides. It means that using the congestion of blood vessels as a sign of inflammation was not possible in most of specimens. Therefore, the disagreement among pathologists will be created when considering blood vessels. In addition, in Cox and Robbins methods, one of the criteria for categorizing the grade of inflammation is vascular congestion that was not discernible and led to misinterpretation of pathologists. On the other hand, using qualitative terms such as scattered, dense and very dense to describe inflammatory cells, led the pathologist to own ideas about inflammation that ending to different interpretation of inflammation grades. In contrast, FDI method mainly focuses on the number of inflammatory cells. In this method, the inflammatory reactions are categorized as 0, none (without inflammatory cells); 1, mild (inflammatory cells ≤25); 2, moderate (25-125 inflammatory cells); 3, severe (more than 125 inflammatory cells) (3). According to this study, three pathologists reached to acceptable agreement when counted the inflammatory cells. In conclusion, it seems that using FDI method is more reliable than Cox method when observing the grades of inflammation in subcutaneous implantation of materials.
